# Protection against Chlamydia Promoted by a Subunit Vaccine (CTH1) Compared with a Primary Intranasal Infection in a Mouse Genital Challenge Model

**DOI:** 10.1371/journal.pone.0010768

**Published:** 2010-05-21

**Authors:** Anja Weinreich Olsen, Michael Theisen, Dennis Christensen, Frank Follmann, Peter Andersen

**Affiliations:** Chlamydia and Adjuvant Research, Department of Infectious Disease Immunology, Statens Serum Institut, Copenhagen, Denmark; Louisiana State University, United States of America

## Abstract

**Background:**

The chlamydial proteins CT443 (OmcB) and CT521 (rl16) have previously been identified as human B and/or T cell targets during a chlamydial infection in humans. Here we compare the protective effector mechanism promoted by a fusion protein composed of CT521 and CT443 (CTH1) with a primary intranasal *Chlamydia muridarum* infection known to provide high levels of protection against a genital chlamydial challenge.

**Methodology/Principal Findings:**

The fusion protein CTH1, adjuvanted with a strong Th1 inducing cationic adjuvant (CAF01), significantly reduced the bacterial shedding compared to a control group in both a *C. trachomatis* Serovar D and *C. muridarum* challenge model. The CTH1/CAF01 vaccine was found to induce polyfunctional T cells consisting of TNFα/IL-2 and TNFα/IL-2/IFN-γ positive cells and high titers of CTH1 specific IgG2a and IgG1. By depletion experiments the protection in the *C. muridarum* challenge model was demonstrated to be mediated solely by CD4^+^ T cells. In comparison, an intranasal infection with *C. muridarum* induced a T cell response that consisted predominantly of TNFα/IFN-γ co-expressing effector CD4^+^ T cells and an antibody response consisting of *C. muridarum* specific IgG1, IgG2a but also IgA. This response was associated with a high level of protection against challenge—a protection that was only partially dependent on CD4^+^ T cells. Furthermore, whereas the antibody response induced by intranasal infection was strongly reactive against the native antigens displayed in the chlamydial elementary body, only low levels of antibodies against this preparation were found after CTH1/CAF01 immunization.

**Conclusions/Significance:**

Our data demonstrate that CTH1 vaccination promotes a CD4^+^ T cell dependent protective response but compared with intranasal *C. muridarum* infection lacks a CD4 independent protective mechanism for complete protection.

## Introduction

Despite the existence of effective antimicrobial therapy, *Chlamydia trachomatis (Ct)* continues to be the leading sexually transmitted bacteria worldwide, causing an estimated 92 million new cases annually [1]. Over 225,000 cases were recorded in 2006 in Europe, making it the most frequently reported infectious disease on the continent [2]. If left untreated, approximately 20% of women with a chlamydial lower genital tract infection will develop pelvic inflammatory disease, 4% will develop chronic pelvic pain, 3% infertility and 2% adverse pregnancy outcomes [3]. Furthermore it has been suggested that *Ct* is a major cofactor for HIV transmission [4] and in the development of cervical neoplasia [5], [6].

Early vaccine trials in humans and non-human primates showed that it was possible to induce protection against *Ct* infection with vaccines based on whole organisms [7], [8]. Nonetheless, as the protection was short lived, and in some trials associated with immunopathology, the approach was abandoned [9]. Thus, identification of potential vaccine antigens and exclusion of potential immunopathogenic components is today an active area of research, one which has been accelerated by the availability of the complete *Ct* serovar D genome sequence [10]. However, so far only relatively few candidates have proven successful in animal models (reviewed in [11]).

Rational vaccine design is based on detailed understanding of pathogen biology and the mechanism leading to protection. Immunity against intracellular pathogens like *Ct* is generally thought to depend on the cellular arm of the immune system. In agreement with this hypothesis, studies in mice and humans have highlighted the importance of CD4^+^ T-helper type 1 (Th-1) cells in the clearance of a chlamydial infection [4], [12], [13]. A number of reports dealing with various pathogens in different animal models have indicated that not only the magnitude but also the quality of the T cell response appears to have significant impact on the establishment of protective memory and protection [14]–[18]. Antibodies have also attracted renewed interest and seem to play an important role during a secondary encounter with *Ct* [19]–[21]. Their role and the exact mechanism of action are however still not clear. In some vaccine studies, antibodies were found largely dispensable [22], whereas studies of vaccines based on the major outer membrane protein (MOMP) found the fine specificity of the humoral response to be critically important [23]. Others have suggested that serum antibodies do not operate through neutralization and that protection against *Ct* requires CD4^+^ T cell subsets in collaboration with antibodies [21], [24].

It has become clear that an effective subunit vaccine against *Ct* must contain multiple epitopes to ensure broad coverage of a genetically heterogeneous population infected with multiple serovars of *Ct* (D to L). The strategy in our laboratory is therefore focused on the molecular engineering of recombinant fusion proteins containing several selected chlamydial vaccine antigens. We have previously demonstrated that fusion proteins can induce amplified responses to molecules with low inherent immunogenicity, leading to significantly higher protection compared to single components or mixtures [25]. The fusion protein approach offers the advantage of a more defined product, reducing the number of recombinant expression and purification steps required. This reduces in turn, the cost of production compared to production and mixing of multiple individual antigenic components.

The purpose of the current study was to evaluate the potential of a subunit vaccine based on the fusion protein CTH1 consisting of the two immunodominant antigens CT443 (omcB) and CT521 (rl16). These antigens were chosen because they represent targets for both arms of the immune system. CT443 is a target for both strong humoral and CMI responses [26] and CT521 was recently discovered by our group to be a strong and frequent target for T cells during natural *Ct* infection in humans [27]. Both of these antigens are highly conserved (>97% homology) across the different serotypes and can be expected to provide substantial levels of cross protection. We examined the immunogenicity and protective efficacy of this fusion protein in comparison to an intranasal (i.n.) *Chlamydia muridarum* (MoPn) infection known to provide a strong protective immune response in the mouse model. CTH1 was combined with the liposomal adjuvant CAF01, known to promote the induction of both CMI and humoral immune responses [28], [29]. Our data demonstrated that although this subunit vaccine induced a high quality protective CD4^+^ T cell response and high levels of CTH1-specific antibodies in both serum and the genital tract, it fails to provide complete protection.

## Results

### Experimental vaccine based on a recombinant fusion protein between CT521 and CT443

**Figure 1:**
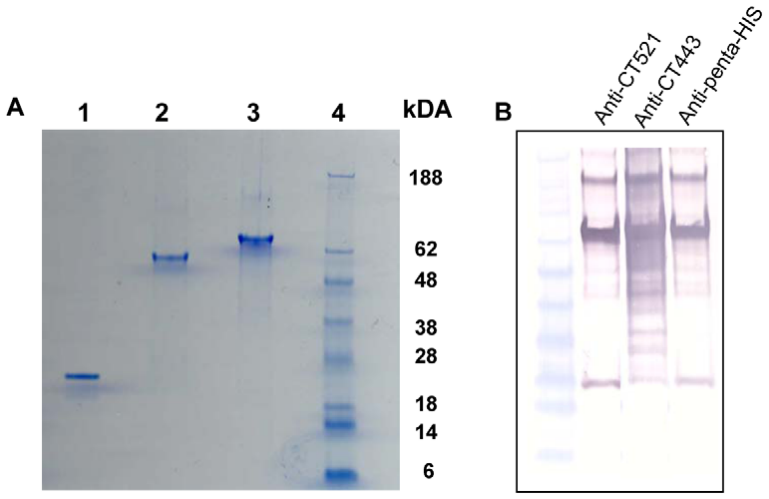
SDS-PAGE and Immunoblotting. A. SDS-PAGE analysis of purified recombinant *Ct* serovar D antigens. 1–1.5 µg of protein was loaded in each lane. Lane 1, CT521; Lane 2, CT443; Lane 3, CTH1; Lane 4, molecular weight standard. Protein bands were visualized by coomassie blue staining. B. Western blot of CTH1 was performed with 1 µg of protein pr. lane. The nitrocellulose membrane was reacted with specific anti-CT521 and anti-CT443 rabbit serum and anti-penta-His (Qiagen, Ballerup, Denmark) (Lane 2, 3 and 4 respectively).

**Figure 2:**
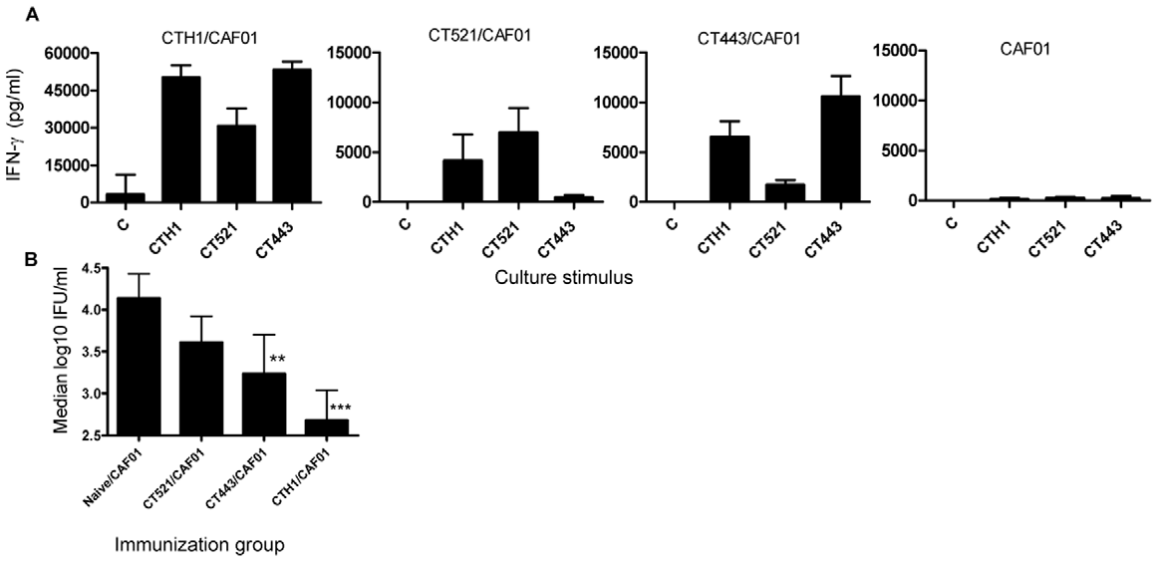
Vaccine-induced immune responses and protection against *C. trachomatis* serovar D. (A) Antigen specific responses by PBMC 5 weeks after the last immunization with CTH1 or the single components CT521 and CT443. C3H/HeN mice were immunized three times at 2 weeks intervals with either CT521 or CT443 or CTH1 emulsified in CAF01. Cell cultures pooled from 13 animals in each group were re-stimulated with 5 µg/ml of CT521, CT443, CTH1and a negative control well (C) without antigen and the IFN-γ responses were measured after 72 h of stimulation. Each point represents the mean of triplicate values±standard deviations. The experiment was performed twice with similar results. (B) Vaccine-induced protection. Six weeks after the final vaccination mice were infected i.vag. with 1×10^7^ IFU of *C. trachomatis* Serovar D/mouse. Median vaginal bacterial loads±interquartil ranges are compared for mice receiving CTH1 (n = 8), CT443 (n = 8), CT521 (n = 8), and controls consisting of naïve and CAF01 immunized mice (n = 16) at day 3 post infection. Culture-negative mice were assigned the lower cut-off of the shedding assay (10 IFU/mouse) **, P<0.01; ***, P<0.001 compared to the control group (Kruskal-Wallis-test, Dunn's posttest).

A fusion protein of CT521 and CT443 (CTH1) was recombinantly expressed in *E. coli*, affinity purified, subjected to ion exchange chromatography, and analysed by sodium dodecyl sulphate-polyacrylamide gel electrophoresis (SDS-PAGE) together with the individual proteins CT521 and CT443 (Fig. 1A). Western blotting demonstrated that CTH1 retained the ability to bind antibodies against both components (Fig. 1B). The initial immunological investigations were done to compare the immunogenicity of the fusion protein with that of the single components and to clarify whether both components of the fusion proteins were recognized by the immune system after immunization. Groups of C3H/HeN mice were immunized subcutaneously three times at two weeks intervals with 5 µg of either the fusion protein or the single components combined with CAF01, an adjuvant known to promote the induction of a highly efficient Th1 and antibody responses [28]–[30]. The immune response to the fusion protein and the single components were investigated five weeks after the last immunization. Immunization with 5 µg of the fusion protein induced a strong IFN-γ release in response to re-stimulation with either the fusion protein or the homologous proteins. Moreover the level of IFN-γ release in response to both CT443 and CT521 restimulation was enhanced in the CTH1 immunized group compared to immunization with the single components (Fig. 2A). Neither mice receiving the adjuvant combination alone (Fig. 2A) nor non-vaccinated naive mice (results not shown) responded to restimulation with CTH1, CT443 and CT521. Six weeks after the last vaccination the mice were challenged intravaginally (i.vag.) with 1×10^7^ inclusion forming units (IFU) of *Ct* Serovar D. Vaginal swab samples were obtained at day 3 and 7 and the protective effects of CTH1/CAF01, expressed as median log_10_ IFU/mouse. Figure 2B shows the difference between the single components and CTH1 at day 3 post infection. CT521/CAF01 did not significantly reduce the vaginal bacterial shedding (P>0.05) whereas mice immunized with CT443/CAF01 had significantly reduced bacterial shedding with a median reduction of 0.9 log10 IFU/mouse (P<0.01). In comparison mice vaccinated with CTH1/CAF01 reduced the bacterial shedding with 1.46 log_10_ IFU/mouse (P<0.001) compared to the control group (from 2.46 log_10_ to 1.0 log_10_). At day 7 post infection there was no significant difference between vaccinated and non-vaccinated mice (results not shown).

### CTH1/CAF01 and MoPn infection induced a different clearance kinetic

**Figure 3:**
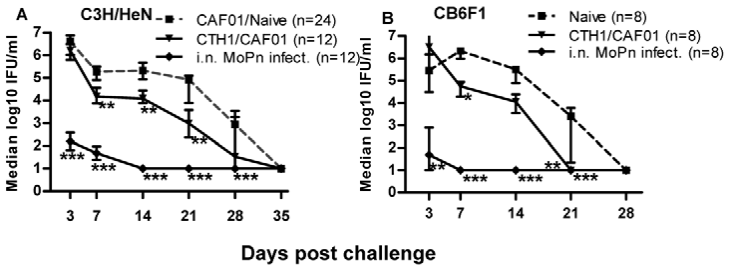
Vaccine-induced protection against MoPn. Mice were either vaccinated 3 times at 2-week intervals with CTH1/CAF01 or i.n infected with 5×10^3^–1×10^4^ MoPn. Six and 8 weeks post last vaccination or infection, respectively mice were i.vag. infected with 1×10^5^ IFU of MoPn. Median vaginal bacterial loads±interquartil ranges at different time points post infection are compared in C3H/HeN mice (A) or CB6F1 mice (B). Culture-negative mice were assigned the lower cut-off of the shedding assay (10 IFU/mouse). * P<0.05, ** P<0.01; *** P<0.001 compared to the control group (Kruskal-Wallis-test, Dunn's posttest).

A *Ct* Serovar D infection is cleared fast in the mouse model resulting in a narrow window for analysing the protective efficacy of a vaccine. Since CT521 is 99% and CT443 97% homologous between *Ct* serovar D and MoPn, we continued by investigating the effect of this vaccine construct in the well established MoPn mouse challenge model. Compared with the brief course of infection with *Ct* serovar D, MoPn is significantly more virulent than *Ct* serovar D and gives rise to a fully developed infection that is cleared between 4–5 weeks post challenge and is associated with disease related pathology [31], [32]. This model therefore allows more detailed studies of the dynamics and mechanisms involved in the vaccine promoted protection and importantly allows a comparison with a well established positive control (prior i.n. infection with MoPn), that provides high levels of protection against a genital challenge [33]. Two different mice strains representing different haplotypes, C3H/HeN (H-2k) and CB6F1 (H-2b and H-2d) were immunized 3 times with 5 µg of CTH1/CAF01 or rendered immune by prior i.n. infection. Six and 8 weeks post vaccination and i.n. infection, respectively, the mice were challenged i.vag. with 1×10^5^ MoPn and vaginal swabs were taken at different time points from day 3 to 35 after challenge infection. Figure 3A and B shows the number of isolated bacteria in the vaginal swabs expressed as median log_10_ IFU/mice in C3H/HeN and CB6F1 mice, respectively. Compared to the control group the CTH1/CAF01 immunized C3H/HeN mice displayed significantly reduced vaginal bacterial shedding at day 7, 14 and 21 post infection (P<0.01) with the most efficient protection expressed at day 14–21 (median IFU/mouse reduced by 1.1–1.9 log_10_) (Fig. 3A). The CTH1 based vaccine also significantly protected CB6F1 mice ranging from 1.5–2.4 log_10_ reduction in IFU's (Fig. 3B). Mice immunized by prior i.n. infection with MoPn cleared the challenge infection in a highly accelerated manner. Whereas CTH1 vaccinated mice had a delay of one week before the first manifestation of protection the i.n. infected group induced a high level of protection immediately after challenge and already at day 7–14 post infection these mice had resolved the infection.

### CTH1/CAF01 subunit vaccination and MoPn infection promotes a different distribution of cytokine producing CD4^+^ T cell subsets

**Figure 4:**
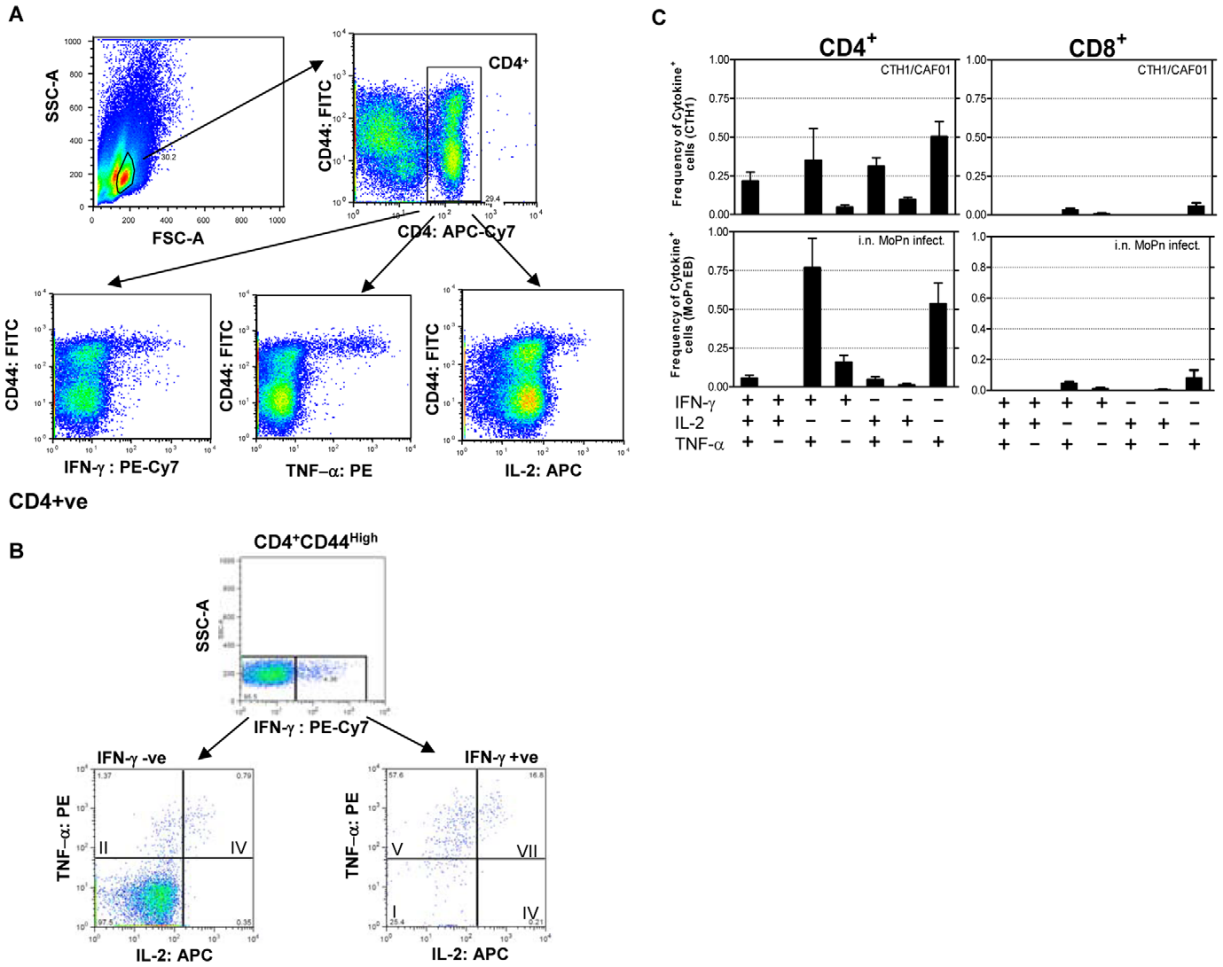
IcFACS analysis of cytokine expression in CD4^+^ and CD8^+^ T cells after CTH1/CAF01 immunization and i.n. MoPn infection. (A) Gating tree for phenotypic and functional characterization of CD4^+^ T cells in CTH1/CAF01 immunised mice by multiparameter flow cytometry. Splenic cells were analysed at 5 weeks after last vaccination or 7 weeks post i.n. MoPn infection by icFACS based on individual spleen cells (4 mice/group) stimulated with either CTH1 or MoPn-EBs. Lymphocytes were gated based on their FSC vs. SSC profile, and the CD4^+^CD44^high^ T cell population was further subdivided into various cytokine-producing subsets. (B) Cytokine-producing cells (IFN-γ, TNF-α, IL-2) within the CD4^+^CD44^high^ population were divided into seven distinct subpopulations based on their production of these cytokines in any combination. (C) Bar chart shows the frequency of each subset within the CD44^high^ CD4^+^ and CD8^+^ T cell populations. The experiment was repeated twice with similar results.

Prophylactic vaccination with CTH1/CAF01 reduced the number of bacteria significantly compared to the control group, but with a lower level of protection and a delayed kinetic compared to a prior i.n. MoPn infection. We therefore characterized and compared the T cell and antibody responses generated by the CTH1/CAF01 vaccine and the i.n. MoPn infection. C3H/HeN mice were vaccinated three times with CTH1/CAF01 or infected i.n. with 5000 IFU of MoPn. As control we included a group of non-vaccinated mice. Five weeks after the last vaccination or 7 weeks after the i.n. MoPn infection, the spleens were isolated from 4 individual mice and splenocytes were stimulated *in vitro* with the homologous antigen preparations, CTH1 and MoPn EBs, respectively. Antigen-specific CD4^+^ and CD8^+^ T cells were characterized based on their ability to secrete the cytokines IFN-γ, TNF-α and IL-2 at the single cell level by intracellular FACS staining. The CD4^+^ and CD8^+^ T cells were analyzed in terms of their CD44 expression and CD44^high^ T cells were assessed for their cytokine-production to establish the proportion of CD4^+^ and CD8^+^ cells positive for IFN-γ, TNF-α and IL-2 (Fig. 4A). The CD4^+^CD44^high^ and the CD8^+^CD44^high^ T cell populations was separated into seven distinct sub-populations based on their production of IFN-γ, IL-2 or TNF-α in any combination and the representation of each of these sub-populations within the pool of T cells established (Fig. 4B). In the CTH1 vaccinated group, multifunctional CTH1-specific CD4^+^ T cells expressing TNF-α/IL-2 (double positive) and IFN-γ/TNF-α/IL-2 (triple positive) CD4^+^ T cells, which has been associated with memory [34], [35], constituted around 1/3 of the total CTH1 specific CD4^+^ T cell population. In comparison the i.n. MoPn infection only induced a very low level of these CD4^+^ T cell subsets and was dominated by CD4^+^ T cells producing IFN-γ/TNF-α (double positive) (Fig. 4C). Neither the vaccine nor the i.n. infection induced detectable numbers of CTH1 and MoPn EB specific CD8^+^ T cells (Fig. 4C) and non-vaccinated mice did not recognize the vaccine components (results not shown).

### CTH1/CAF01 mediated protection is CD4^+^ T cell dependent in the MoPn model

**Figure 5:**
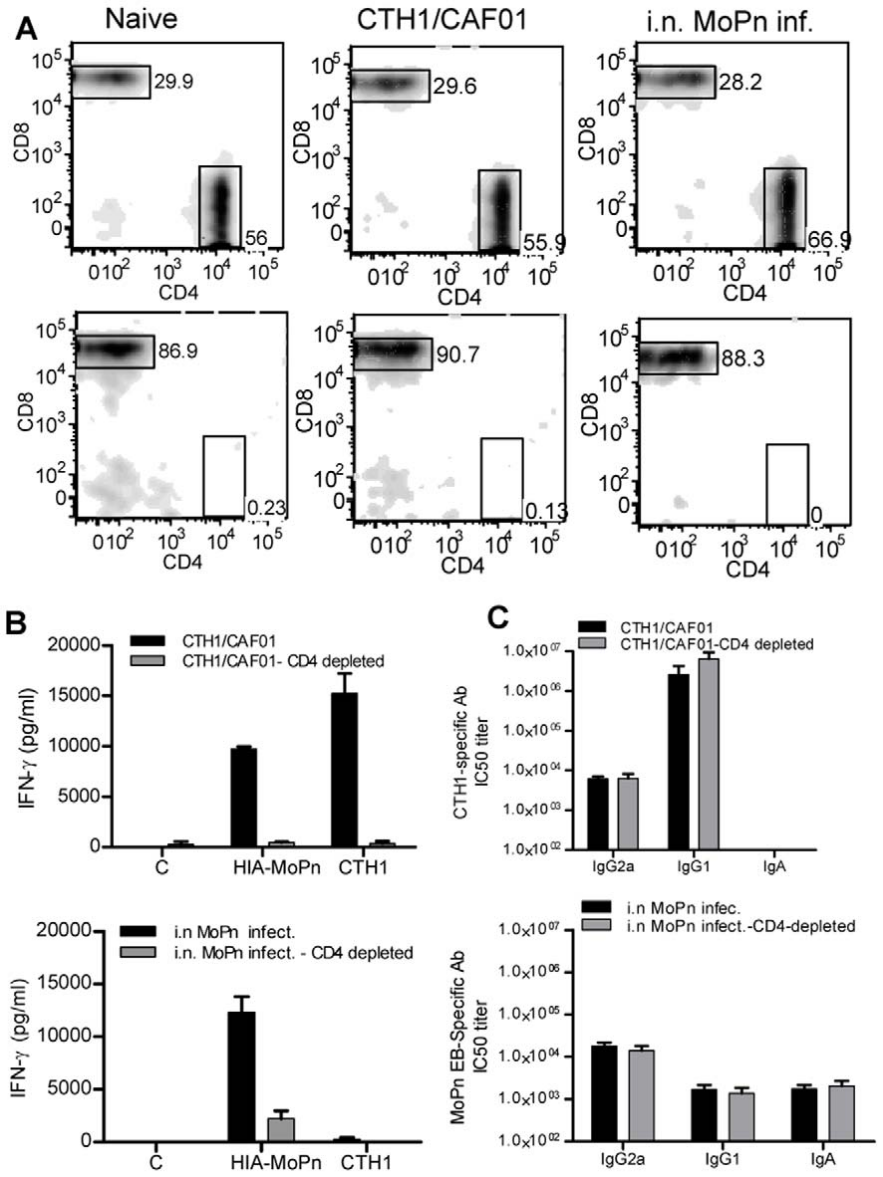
Immune responses of CD4-depleted and non-depleted mice. (A) The density plot represents CD4^+^ and CD8^+^ T cells in PBMCs 2 days post challenge in non-depleted (top panel) and CD4-depleted mice (lower panel). (B) CTH1-specific and MoPn-specific IFN-γ responses in depleted and non-depleted CTH1 vaccinated and i.n. MoPn infected C3H/HeN mice. The IFN-γ responses were measured in triplicate cell cultures pooled from 3 animals/group 2 days post challenge. Each group was re-stimulated with 5 µg/ml of CTH1 and HIA-MoPn and IFN-γ responses were measured after 72 h of stimulation. Each bar represents the mean of triplicate values±standard deviations, (C) CTH1-specific and MoPn-specific antibodies generated after CTH1 vaccination or i.n. MoPn infection in CD4 depleted and non-depleted mice Antibodies (IgG1, IgG2a and IgA) were measured in serum (diluted 5 fold from a 1:20 dilution) obtained 2 days after challenge infection, by ELISA. Reciprocal serum dilutions corresponding to 50% maximal binding (EC50) were computed using the Prism 4 software. Each bar represents the mean EC50±SEM of 3 individual mice. The experiment was repeated twice with similar results.

**Figure 6:**
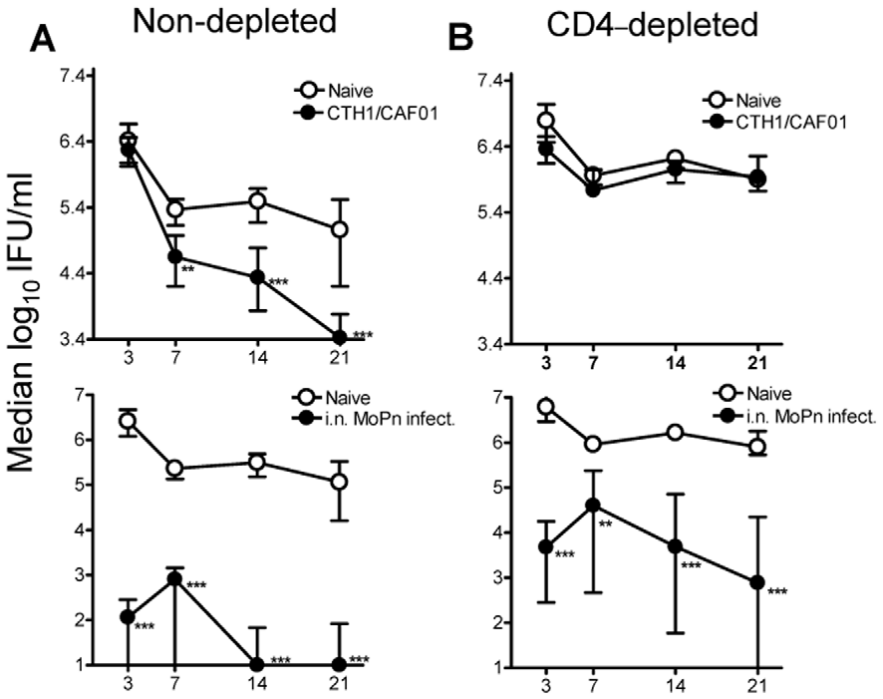
Importance of CD4^+^ T cell immunity in vaccine-mediated protection. C3H/HeN mice depleted of CD4^+^ T cells received i.p. injections of 250 µg of anti–mouse CD4 monoclonal antibody (clone GK1.5) on days -7, -4, -3 and of 400 mg purified antibody at day +1, +4, +8, +11, +15,+18,+22 relative to the day of infection. Mice were vaccinated and challenged as described for Fig. 2. Swab samples were obtained on days 3, 7, 14, 21 and 35 post infection. Median vaginal MoPn loads±interquartile ranges were compared for (A), top panel: control mice (n = 20) vs. CTH1/CAF01 vaccinated mice (n = 16). (A), lower panel: control mice (n = 20) vs. i.n. MoPn infected mice (n = 8) (B), top panel CD4-depleted control mice (n = 8) vs. CD4-depletion CTH1/CAF01 immunized mice (n = 8). (B), lower panel: CD4-depleted control mice (n = 8) vs. CD4-depleted i.n. MoPn infected mice (n = 8). Culture-negative mice were assigned the lower cut-off of the shedding assay (10 IFU/mouse). **, P<0.01; *** P<0.001 compared to the control group (Kruskal-Wallis-test, Dunn's post test).

We continued by investigating the contribution of the CD4^+^ T cells in the protective immune response promoted by CTH1/CAF01 and i.n. MoPn infection. C3H/HeN mice were vaccinated 3 times with CTH1/CAF01 or infected once i.n. with 5000 IFU of MoPn. A non-vaccinated group was included as control. At three time points before challenge (Day -7, -5 and -3), 8 mice from each group were depleted of CD4^+^ T cells through injection of monoclonal anti-mouse CD4 antibodies (GK 1.5). Injection of a relevant rat IgG2b (isotype control) in a separate group had no impact on the number of CD4^+^ T cells (data not shown). Six weeks after the last immunization with CTH1/CAF01 or 8 weeks after the primary i.n. MoPn infection the mice were challenged i.vag. with 10^5^ MoPn and vaginal swabs were taken at day 3, 7, 14, and 21 post infection. The depletion of CD4^+^ T cells was continued by injections of GK.1.5 every 3–4 days (Day +1, +4, +8, +11, +15, +18 and +22). FACS analysis on the blood 2 days after challenge showed that the CD3^+^/CD4^+^ cell population of the total CD3 population was between 0–0.23% in mice receiving GK1.5 compared to approximately 60% in non-depleted mice, indicating a very efficient depletion of the CD4^+^ T cell subset in treated animals (Fig. 5A) which was maintained throughout the infection period. The absence of CD4^+^ T cells was also seen in collagenase treated genital tract tissue of depleted mice (Figure S1). In agreement with this, CD4-depleted CTH1/CAF01 immunized and i.n. MoPn infected mice only induced marginal IFN-γ responses to CTH1 or MoPn EBs, respectively compared to the non-depleted mice (Fig. 5B) The CD4-depletion had no effect on the level of specific antibody to neither CTH1 nor MoPn EB (Fig. 5C). CTH1 immunization induced high levels of IgG1 and IgG2a but no IgA, whereas the i.n MoPn infected mice induced high levels of all three types. Analysis of the vaginal bacterial shedding showed that the protection promoted by CTH1/CAF01 vaccination (Fig. 6A, top panel) was completely eliminated after CD4^+^ T cell depletion (Fig. 6B, top panel), suggesting that the protection from CTH1/CAF01 vaccination is solely CD4^+^ T cell dependent. In agreement with earlier observations (Fig. 3A) mice i.n. infected with MoPn reduced the level of bacterial shedding with 4 log_10_ as early as day 3 post challenge infection (Fig. 6A, lower panel). Depleting CD4^+^ T cells in this group resulted in impaired resistance and 1.5–3 log more bacteria throughout infection demonstrating a clear involvement of CD4^+^ T cells also in the protection promoted by prior i.n. infection with MoPn (Fig. 6B, lower panel). It was on the other hand clear that even in the absence of CD4^+^ T cells, bacterial shedding was reduced with around 3 log_10_, compared to the non-immunized group which suggests an effector mechanism independent of CD4^+^ T cells in the i.n. immunized group.

### CTH1/CAF01 induced high levels of specific antibodies but with limited binding potential for the chlamydial EBs

**Figure 7:**
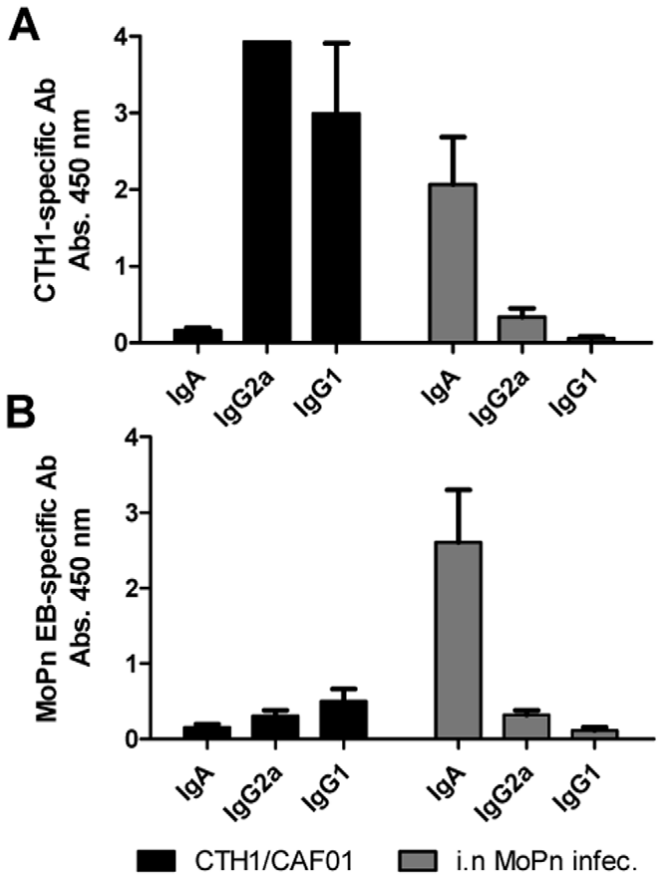
CTH1-specific (A) and MoPn-specific (B) antibodies generated after vaccination or i.n MoPn infection. Mice were either immunized with CTH1/CAF01 or infected i.n. with MoPn. Non-immunized control mice were included as controls. Antibodies (IgG1, IgG2a and IgA) were measured in vaginal secretions (dilution shown 1:10) obtained 8 days after challenge infection, by ELISA. Each symbol and bar represents the mean Abs 450±SD of 4 individual mice.

We finally compared the capacity of antibodies generated after CTH1/CAF01 vaccination and i.n. MoPn infection to bind to the MoPn EBs. Vaginal secretions from CTH1/CAF01 immunized, i.n. MoPn infected, and control animals were tested by ELISA coated with CTH1 and chlamydial EBs. We found that CTH1/CAF01 immunization induced very high levels of CTH1-specific IgG1 and IgG2a in the vaginal secretions (Fig. 7A, black bars). However, these CTH1/CAF01 specific antibodies only had minimal ability to bind MoPn EBs (Fig. 7B, black bars) or *Ct* serovar D (results not shown). No CTH1 or MoPn EB specific IgA could be detected after CTH1/CAF01 immunization. In contrast i.n MoPn infection was associated with high levels of IgA in the vaginal secretions directed to both CTH1 and MoPn EB (Fig. 7A and 7B (gray bars)).

## Discussion

T helper-type 1 (Th1)-type CMI responses play a predominant role in anti-chlamydial protective immunity [12], [13], [36]. Identification of antigens recognized by CD4^+^ T cells [37]–[40] and delivery of these antigens to induce a strong CMI response is therefore a central goal when developing novel vaccines against *Ct*. In the present study we have genetically engineered a fusion molecule encoding two chlamydial proteins; CT521 that resides in the cytosol of reticulate bodies during the replicating cycle (data not shown) and CT443 (omcB) located in the outer cell wall [41], [42]. Both proteins are frequently recognized by T cells from patients with a confirmed genital chlamydial infection [26], [27] and CT443 is, in addition, a well known antibody target [26], [43], [44].

The CTH1 antigen was delivered in CAF01, an adjuvant system, known to induce both strong CMI and antibody response [28]–[30]. Our study demonstrates that this vaccine promotes significant protection against genital infection in mice infected with either *Ct* serovar D or MoPn. *Ct* serovar D has relatively low virulence in the mouse and gives rise to a transient infection. The protection promoted by the vaccine was therefore evident at day 3 post infection, but by day 7 the window between vaccinated and control animals became too narrow to detect any significant difference. Since the *Ct* Serovar D sequence of the CTH1 protein is 97% homologous to the MoPn sequence of CTH1 we decided to investigate the protective capacity of CTH1 using MoPn as challenge strain. Compared to the *Ct* serovar D challenge model we found the kinetic of bacterial growth and elimination to be different. There was no initial reduction in IFU in vaccinated animals, but from day 14–21 we could detect up to a 2 fold reduction in IFUs compared to a control group. The level of protection and the delayed clearance was not due to a lack of strain specific immunity as later experiments have demonstrated similar infection dynamics after vaccination with a MoPn version of CTH1 (results not shown). Importantly, the CTH1 mediated reduction of IFU was found to be mediated exclusively by CD4^+^ T cells.

CMI-induced protection has been observed in other recent studies promoted by subunit vaccines based on CPAF or NrdB in which IL-12 or CpG and cholera toxin plus CpG were used as adjuvants, respectively. For both of these vaccines, protection was found to be dependent upon CD4^+^ T cells producing IFN-γ [38], [45], [46]. IFN-γ has often been used as a single readout for Th1 responses, but recent studies of other infectious diseases have emphasized the importance of polyfunctional T cells co-producing IL-2, IFN-γ and TNF-α [14], [47]. Here we demonstrated that CTH1/CAF01 immunized animals had a high percentage of antigen specific CD4^+^ T cells co-expressing IFN-γ/TNF-α/IL-2 or TNF-α/IL-2. IL-2 co-expression has been demonstrated to play an important role in the long-term survival of primed CD4^+^ T cells *in vivo* [34], [35]. Furthermore these polyfunctional cells have been demonstrated to correlate with efficient protection against other intracellular pathogens such as *Leishmania major* and *Mycobacterium tuberculosis* [14], [48]. In the present study, the proportion of polyfunctional T cells are much higher in the CTH1/CAF01 immunized group compared to the i.n. MoPn infected group. This suggests that the CTH1 vaccine promotes a fully-adequate, high quality T cell response, as do other vaccines employing the liposome based adjuvant CAF01 [48]. It is therefore likely that the explanation for the lower levels of protection promoted by the subunit vaccine should be sought elsewhere than in the quality of the CMI response induced. In agreement with this, our data clearly demonstrate that whereas the protection in the subunit vaccinated mice was solely dependent on CD4^+^ T cells, the i.n. MoPn infected mice displayed a very substantial protective immune response even in the absence of CD4^+^ T cells.

The CD4^+^ T cell independent effect is most likely not mediated by CD8^+^ T cells as CD8-depletion of i.n. infected mice had minimal influence on the level of protection (authors' unpublished observations). This is in agreement with other observations from mouse models of genital tract infection where immunity is neither diminished in the absence of CD8^+^ T cells nor is any significant protection conferred by CD8^+^ T cells [12], [49], [50]. Another T cell subset that has been demonstrated to play a potential role in the immune response to intracellular pathogens is γδ T cells. Similarly to CD4^+^ and CD8^+^ T cells, γδ T cells secrete IFN-γ, can lyse infected macrophages and can help contain bacterial growth (reviewed in [51]). Their role in Chlamydia infection, although still not completely resolved, seems to be very modest [13], [52] and is therefore most likely not responsible for the CD4 independent protection we observe in the present study. However, a number of important studies from Morrison and colleagues have indicated a previously unrecognized protective role for antibodies and B cells against secondary genital chlamydial infection in mice [20], [21], [50], [53]. The mechanisms of action for antibodies in immunity to Chlamydia remain unresolved and data from different laboratories collectively suggest a more complex role than just direct neutralisation of the invading pathogen. Mice deficient in activating Fc receptors (FcR−/− mice) had impaired resistance to secondary chlamydial challenge [24], [54]. This has been interpreted to suggest that the effects of antibody may occur via Fc receptor-dependent mechanisms that could accelerate the subsequent CMI response. That CMI and antibodies interact in protection against Chlamydia was also emphasized by the finding that antibody-mediated protection is highly dependent on CD4^+^ T cell-mediated adaptive changes that occur in the local genital tract tissues during primary infection [53]. Our study demonstrates that CTH1/CAF01 vaccination induced high levels of CTH1 specific IgG1 and IgG2a both in blood and vaginal secretions. However, the antibodies were only weakly reactive to chlamydial EBs of either MoPn (Fig. 7) or *Ct* serovar D. In contrast, mice i.n infected with MoPn induced high levels of IgA directed to EBs in both serum and vaginal wash. There are at least two explanations for this difference. It may relate to the recognition of multiple target antigens on the surface of EB in the i.n. vaccinated group as demonstrated elsewhere [55], compared to the focused response in CTH1 vaccinated animals directed to CT443 and CT521. The weak binding of CTH1-specific antibodies to the whole EB preparation could be explained by a relatively low concentration of CT443 and CT521 in the ELISA wells of EB coated plates compared to CTH1 coated plates. Alternatively, the weak recognition of the EB preparation in the CTH1 vaccinated mice may relate to differences in the conformation of the two vaccine antigens in their natural localization in EB's compared to the recombinant vaccine construct. CT443 (OmcB) was originally identified as part of the outer membrane complex [41], and recent data have demonstrated that this molecule functions as a chlamydial adhesin [42], emphasizing its surface localization. CT443 may however suffer from the same problem as MOMP, where a number of recent studies have demonstrated that the optimal antibody response and protective efficacy depends on its native conformation-something that has so far not been possible to achieve with recombinantly expressed MOMP [23], [56].

In conclusion, our data suggest that the efficient protection promoted by i.n. infection is mediated partly by a CD4 independent mechanism. Our CTH1 construct efficiently induces both CD4 T cell and a surface targeted antibody response. While the CTH1 vaccine obviously needs further improvement this will not necessarily be achieved by the incorporation of additional antigens. CTH1 may face the same hurdles as often encountered in attempts to recombinantly produce biologically active proteins, where refolding into bioactive forms is cumbersome, results in poor recovery and accounts for the major cost and complexity. For a fusion molecule like CTH1 which incorporates the cysteine rich CT443, restoration of the native-like secondary structure or at least exposure of important linear B cell epitopes will obviously be very difficult and demand carefully targeted mutagenesis of selected cysteine residues. Work towards this goal is currently ongoing in our laboratory.

## Materials and Methods

### Ethics Statement

The handling of mice was conducted in accordance with the regulations set forward by the Danish Ministry of Justice and animal protection committees by Danish Animal Experiments Inspectorate, and in compliance with European Community Directive 86/609 and the U.S. Association for Laboratory Animal Care recommendations for the care and use of laboratory animals. All the techniques/procedures have been refined to provide for maximum comfort/minimal stress to the animals.

### Organisms

The *C. muridarum* strain MoPn/NiggII and *C. trachomatis* serovar D was purchased from the ATCC and propagated in HeLa-229 cells as described previously [27]. Chlamydial EBs were harvested, purified and quantified as described previously [27], [30] and stored at −80°C in a 0.2 M sucrose, 20 mM sodium phosphate and 5 mM glutamic acid buffer (SPG).

### Cloning, gene expression and protein purification of recombinant proteins for vaccination

#### Cloning of vaccine hybrid construct CTH1

DNA fragments containing the genes of *ct521* and *ct433* were amplified from *Ct* serovar D genomic DNA by overlap extension PCR [57]. Amplifications were carried out for 25 cycles each with denaturation at 94°C for 30 sec, annealing at 55°C for 30 sec, and extension at 72°C for 2 min, using Phusion polymerase (Finnzymes, Espoo, Finland). Nucleotide sequencing was performed directly on the PCR products by MWG-Biotech AG (Germany) using specific sequencing primers. The *ct521*-*ct433* gene fusion was created using the specific primer Ct521_fw_1 (5′- CAC CGG ATC CAT GTT AAT GCC TAA ACG AAC AAA ATT TC and Ct521_rev_1 (5′- CAC CCC GCT AGC AAA TAA ACT TAC CCT TTC CAC ACG CTT AAC AAA) [*ct521*], Ct443_fw_1 (5′-TTT GTT AAG CGT GTG GAA AGG GTA AGT TTA TTT GCT AGC GGG GTG) and Ct443_rev_1 5′-GGA TCC CTA ATA GAT GTG TGT ATT CTC TGT ATC AGA AAC TG [*ct433*] in a first round PCR using chlamydial DNA extracted as described in [58] as the template. The respective products were used as templates in second round PCR using the primers Ct521_fw_1 and Ct443_rev_1. The resulting DNA fragment was cloned into pENTR/D-TOPO and subsequently into pDEST17 expression vector (Invitrogen, Copenhagen, Denmark) thereby creating an in frame fusion with 6*His tag.

#### Recombinant gene expression and protein purification

*Escheria coli* BL-21 AI cells transformed with plasmid pDEST17 (Invitrogen, Copenhagen, Denmark) encoding CTH1 were grown at 37°C to reach the logarithmic phase OD_600_ ∼0.5 and protein expression was induced by adding arabinose to total concentration of 0.2%. The protein expression was induced for 4 hours and cells were harvested by centrifugation (6,000 g for 15 min.). *E. coli* were lysed using Bugbuster (Novagen, Darmstadt, Germany) containing Benzonase, rLysozyme and Protease inhibitor Cocktail I (Calbiochem, San Diego, CA) to avoid unwanted degradation. Lysis was performed at room temperature for 30 min. during gentle agitation. Inclusion bodies were isolated by centrifugation (10,000 g for 10 min.) The pellet was washed once with 1∶5 diluted Bugbuster solution in 3 M urea and then dissolved in 50 mM NaH_2_PO_4_, 0.4 M NaCl, 8 M Urea, 10% glycerol, 10 mM Imidazole pH 7.5. This solution was loaded onto a 5 ml HisTrap HP (Amersham Biosciences, Buckinghamshire, United Kingdom) and the bound proteins were eluted by applying a gradient of 50 to 500 mM imidazole. Fractions containing the desired recombinant protein were pooled, dialyzed against 20 mM ethanolamine, pH 9, 8 M urea and applied to a 5 ml HiTrap Q Sepharose HP (Amersham Biosciences, Buckinghamshire, United Kingdom). The recombinant protein was eluted by applying a gradient of 0 to 1 M NaCl over 10 column volumes. Analysis of all fractions was performed by SDS-PAGE. Protein concentrations were measured by the BCA protein assay (Pierce, Rockford, Illinois, USA). The purity was assessed by SDS-PAGE followed by coomassie staining and western blot with anti-penta-His (Qiagen, Ballerup, Denmark) and anti-*E. coli* antibodies to detect contaminants (DAKO, Glostrup, Denmark). CTH1 was refolded by a stepwise removal of buffer containing urea ending up in 20 mM Citrate-phosphate buffer pH 4, 10% glycerol, 1 mM cysteine which yielded soluble protein.

Recombinant gene expression and protein purification of CT443 and CT521 was done as described in [26]. The purified recombinant proteins were stored at −20°C until use.

### SDS-page and Immunoblotting

SDS-PAGE and western blot of CT521, CT443 and CTH1 was performed with 1 µg of protein pr. lane (Figure 1 A and B). The nitrocellulose membrane was reacted with specific anti-CT521, anti-CT443 rabbit serum and anti-penta-His (Qiagen, Ballerup, Denmark) (Lane 2, 3 and 4 respectively).

### Immunizations

Female 6–8 weeks old C3H/HeN and CB6F1 mice were purchased from Harlan Scandinavia (Denmark). Animals were immunized subcutaneously with 1–5 µg/dose of CT521, CT443 or CTH1 in 100 µl sterile Tris-buffer (pH 7.4) mixed by vortexing with 100 µl CAF01 adjuvant (SSI) consisting of 50 µg/dose of the glycolipid trehalose 6,6′-dibehenate (TDB) incorporated into 250 µg/dose of cationic liposomes composed of dimethyldioctadecylammonium (DDA) bromide [28]. Mice received a total of three immunizations at two week intervals. Ten and 3 days before MoPn or *Ct* serovar D challenge, the oestrus cycle was synchronized by injection of 2.5 mg Medroxyprogesteronacetat (Depo-Provera; Pfizer). Six weeks after the final vaccination the mice were challenged i.vag. with 1×10^5^ IFU of MoPn or 1×10^7^ IFU of *Ct* serovar D in 10 µl SPG buffer. For i.n. infection with MoPn, mice received from 5×10^3^ to 1×10^4^ bacteria 8 weeks prior to i.vag. challenge. Specific antisera against CT443 and CT521 were generated by injecting recombinant proteins 50 µg into rabbits in combination with Montanide ISA720. Two booster doses were administered at 2-week intervals.

### Depletion of CD4^+^ T-cells

Monoclonal anti-mouse CD4 IgG2b (clone GK1.5) was purified from hybridoma supernatants made in our lab, using HiTrap protein G HP columns (Amersham Biosciences). The purified IgG was dialyzed against PBS, sterile filtered and the protein concentration was determined. Mice were depleted of CD4^+^ T-cells by the i.p route with 3 injections of 250 µg purified antibody each at day -7, -5, -3 followed by 400 µg purified antibody at day +1, +4, +8,+11,+15,+18,+22 relative to the day of infection. The depletion of CD4^+^ T-cells was verified by FACS analysis on PBMCs at day, +2, +7, and day +16 relative to the day of infection using a FITC conjugated anti-CD4 antibody (clone RM4-4), a PE conjugated anti-CD8 antibody (clone 53-6.7) and a APC conjugated anti-CD3 (clone 145-2C11)(BD Biosciences). Likewise, genital tract tissue was removed collagenase (0.7 mg/ml)/DNAse (40 µg/ml) treated for 1 h and surface stained for FACS analysis. Injection of isotype control antibody was found to have no impact on the size of the CD4^+^ T-cell population.

### Measurement of antibody levels in serum and vaginal secretions

Blood and vaginal secretion fluid were collected for quantification of vaccine-specific antibodies by ELISA. Blood were collected from the periorbital vein plexus and centrifuged 2500 c.p.m. to separate serum. Vaginal secretion samples were collected by flushing the vagina with 100 µl of sterile PBS. Maxisorb Plates (Nunc, Roskilde, Denmark) were coated with either CTH1 (1 µg/ml) or heat inactivated (HIA) MoPn EBs (10 µg/ml) and the samples were serially diluted before added to ELISA-plates. Serum samples and vaginal secretion fluid were 5 fold diluted 8 times from a 1∶20 and 1∶10 dilution, respectively. Antigen-specific IgG1, IgG2a, and IgA were detected with isotype-specific HRP-conjugated rabbit anti-mouse IgG (Zymed) diluted 1∶5000. Substrate was TMB-PLUS (Kem-En-TEC, Taastrup, Denmark). Reciprocal serum dilutions corresponding to 50% maximal binding (EC50) were computed using the GraphPad Prism 4.

### Chlamydia-specific cellular responses

Blood lymphocytes were purified on a density gradient. Cells were pooled from 3–13 mice in each group. Single-cell suspensions were prepared from individual spleens (4 mice/group) and obtained by homogenisation through a metal mesh and washed twice in RPMI-1640 (Gibco Invitrogen, Taastrup, Denmark). All cell cultures were grown in Nucleon microtiter plates (Nunc, Roskilde, Denmark) containing 2×10^5^ cells/well in 200 µl RPMI-1640 supplemented with 5×10^−5^ M 2-mercaptoethanol, 1 mM glutamine, 1% pyruvate, 1% penicillin-streptomycin, 1% HEPES and 10% fetal calf serum (FCS) (Invitrogen, Taastrup, Denmark). The cells were restimulated with either HIA-MoPn (5 µg/ml), CTH1 (5 µg/ml), CT443 (5 µg/ml) or CT521 (5 µg/ml). Stimulation with Concavalin A (5 µg/ml) (results not shown) or media (C) were done as positive control for cell viability and negative control, respectively. After 72 h of incubation at 37°C/5% CO_2_, supernatants were harvested and stored at −20°C before use. The amounts of secreted IFN-γ were determined by enzyme-linked immunosorbant assay (ELISA) as described elsewhere [59].

### Intracellular cytokine staining procedure

Splenocytes were stimulated over night with 5 µg/ml of CTH1 or HIA-MoPn at 37°C/5% CO_2_ and subsequently incubated for 5 h at 37°C with 10 µg/ml brefeldin A (Sigma-Aldrich, USA) and 0.7 µl/ml monensin/GolgiStop (BD Pharmingen). The cells were washed in FACS buffer (PBS containing 0.1% sodium azide and 1% FCS) before surface staining with rat anti-mouse antibodies. The intracellular cytokine staining procedure was done essentially as described in [48]. Cells were washed with FACS buffer before fixation and permeabilization using the BD Cytofix/Cytoperm™ (BD, San Diego, CA, USA). The following antibodies were used for surface staining: PerCP-Cy.5.5-anti-CD8α (53-6.7), APC-Cy7-anti-CD4 (GK1.5), and FITC-anti-CD44. The following antibodies were used for intracellular staining: PE-anti-TNF-α, APC-anti-IL-2, PE-Cy7-anti-IFN-γ. All antibodies were purchased from BD Pharmingen (San Diego, USA) or eBiosciences (San Diego, USA).

### Vaginal chlamydial load

Vaginal swabs were obtained at 3, 7, 14, 21, 28 and 35 days after infection. Swabs were vortexed with glass-beads in 1 ml SPG buffer and stored at −80°C until analysis. Infectious load was determined as described in [30]. Briefly, McCoy cell monolayers were infected with a titrated volume of the swab suspension. The plates were centrifuged at 750×g for 1 h at RT followed by incubation at 35°C for 2 h. Infection-media was then replaced with fresh media and the cells incubated at 37°C for 30 h. Inclusions were visualised by staining with polyclonal rabbit anti-MOMP serum made in our laboratory, followed by a FITC conjugated swine anti-rabbit Ig (DAKO, Glostrup, Denmark). Background staining was done with propidium iodide (Invitrogen, Taastrup, Denmark). Inclusions were enumerated by fluorescence microscopy observing at least 20 individual fields of vision for each well.

### Statistical analysis

Statistical analysis was done using GraphPad Prism 4. Medians of vaginal chlamydial load were analyzed using Kruskall-Wallis followed by Dunn's post test. A value of P<0.05 was considered statistically significant.

## Supporting Information

Figure S1 Genital tract tissue (representative of 4 individually mice) was isolated from CD4- depleted and non-depleted i.n MoPn infected mice and surface stained using a FITC conjugated anti-CD4 antibody (clone RM4-4), a PE conjugated anti-CD8 antibody (clone 53-6.7) and a APC conjugated anti-CD3 (clone 145-2C11) antibody.
(0.04 MB TIF)
