# Supplementary figures and images for: Protection against Chlamydia Promoted by a Subunit Vaccine (CTH1) Compared with a Primary Intranasal Infection in a Mouse Genital Challenge Model
Source: PLoS One. 2010 May 21;5(5):e10768. doi: 10.1371/journal.pone.0010768 (PMC2874006; doi:10.1371/journal.pone.0010768)

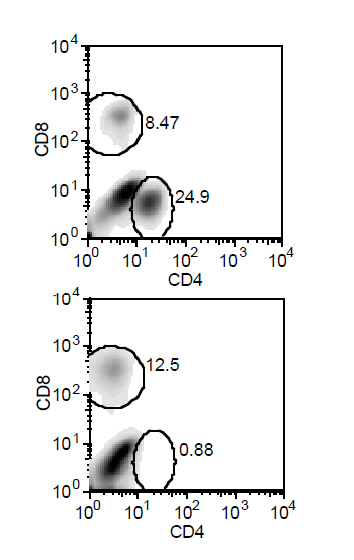

Supplement: Figure S1 — Genital tract tissue (representative of 4 individually mice) was isolated from CD4- depleted and non-depleted i.n MoPn infected mice and surface stained using a FITC conjugated anti-CD4 antibody (clone RM4-4), a PE conjugated anti-CD8 antibody (clone 53-6.7) and a APC conjugated anti-CD3 (clone 145-2C11) antibody. (0.04 MB TIF) [file pone.0010768.s001.tif]
